# Wearable-Sensors-Based Platform for Gesture Recognition of Autism Spectrum Disorder Children Using Machine Learning Algorithms

**DOI:** 10.3390/s21103319

**Published:** 2021-05-11

**Authors:** Uzma Abid Siddiqui, Farman Ullah, Asif Iqbal, Ajmal Khan, Rehmat Ullah, Sheroz Paracha, Hassan Shahzad, Kyung-Sup Kwak

**Affiliations:** 1 Electrical and Computer Engineering Department, COMSATS University Islamabad Attock Campus, Attock 43600, Pakistan; uzmaabidsiddiqui@gmail.com (U.A.S.); drajmal@ciit-attock.edu.pk (A.K.); sheryparacha666@gmail.com (S.P.); hshahzad837@gmail.com (H.S.); 2 Department of Information and Communication Engineering, Inha University, Incheon 22212, Korea; asifsoul@inha.ac.kr; 3 Department of Computer Systems Engineering, University of Engineering and Technology, Peshawar 25000, Pakistan; rehmatkttk@nwfpuet.edu.pk

**Keywords:** wearable sensors, autism spectrum disorder (ASD), stereotype movements, gestures, machine learning, KNN, decision tree, random forest, neural network

## Abstract

Autistic people face many challenges in various aspects of daily life such as social skills, repetitive behaviors, speech, and verbal communication. They feel hesitant to talk with others. The signs of autism vary from one individual to another, with a range from mild to severe. Autistic children use fewer communicative gestures compared with typically developing children (TD). With time, the parents may learn their gestures and understand what is occurring in their child’s mind. However, it is difficult for other people to understand their gestures. In this paper, we propose a wearable-sensors-based platform to recognize autistic gestures using various classification techniques. The proposed system defines, monitors, and classifies the gestures of the individuals. We propose using wearable sensors that transmit their data using a Bluetooth interface to a data acquisition and classification server. A dataset of 24 gestures is created by 10 autistic children performing each gesture about 10 times. Time- and frequency-domain features are extracted from the sensors’ data, which are classified using k-nearest neighbor (KNN), decision tree, neural network, and random forest models. The main objective of this work is to develop a wearable-sensor-based IoT platform for gesture recognition in children with autism spectrum disorder (ASD). We achieve an accuracy of about 91% with most of the classifiers using dataset cross-validation and leave-one-person-out cross-validation.

## 1. Introduction

Autism spectrum disorder, commonly called autism, is defined as a variety of disorders, which include challenges with social rules, difficulty in verbal and non-verbal communication, and restricted or repetitive actions [1]. Each child with ASD has their own specific needs and a collection of habits and behaviors that can hinder their day-to-day tasks. As ASD is a heterogeneous neurodevelopmental disorder, its symptoms appear during the early ages, normally in the first two or three years of life [2]. It is an intricate neurobehavioral condition that makes social interactions problematic for such individuals. Not all the disorders in this spectrum are equally severe; some appear to be a minor handicap, while others are serious and can practically disrupt the whole lifestyle of the affected person. Children with ASD struggle to communicate with others. Reading visual emotions is tricky for them and they usually struggle to understand what other people feel and think. 

Autistic people face many challenges in their daily lives in areas such as social skills, repetitive behaviors, speech, and nonverbal communication, and experience feelings of hesitation. They use fewer communicative gestures compared with typically developing children (TD), so they struggle to convey their ideas or thoughts with words, gestures, or facial expressions. Usually, people with autism develop strange behaviors and, in some cases, they can be dangerous to themselves and to the people around them [3]. Due to the impairment in their speaking ability, their children’s physical abilities may be weakened, which can lead to risk [3]. These disorders are more common in boys than girls, with a ratio of about 4:1 [4]. To effectively communicate with people from an early age and initiate social interactions, the child must be able to understand verbal and non-verbal messages [5]. Gestures are the universal and most important feature of our communication [6]. Verbal gestures involves the use of our voice and co-verbal gestures involve hand and arm movements. They simplify linguistic content, emphasize our point of view, regulate the flow of speech, and maintain the audience’s attention toward the speaker. Although there is no rigid rule about gesture categories, conventional gestures (CG) have well-established premises [7]. These are conversational and deliberate actions that enable direct and accurate verbal translation so that they are easily understood even without spoken help. Because conventional gestures contribute to communication and are a pre-cursor to verbal language, their recognition in children presenting neurodevelopmental disorders is crucial. Over the course of time, their parents can grow accustomed to their gestures and understand what is occurring in their child’s mind, but others may find these gestures difficult to interpret.

Due to nonverbal communication or repetitive speaking, ASD children have difficulties conveying their message and other people struggle to understand their gestures. Sign-language, or hand-speak, has become a popular method of communicating for those who cannot verbally speak. However, sign language is difficult to understand for normal people. A gesture translator is needed to communicate with ASD people. The Internet of Things (IoT) is providing new and emerging capabilities through continuous connectivity. This concept generally revolves around the rapidly expanding environments of computing capabilities and network connectivity to sensors, objects, devices, and items that efficiently exchange digital data with each other without external assistance, i.e., human involvement. The main objective of the IoT is to design, operate, deploy, and transform physical devices through data acquisition, predictive analytics, smart networking, deep optimization, and some other related solutions [8]. Wearable devices provide opportunities for innovative services in health sciences along with predictive health monitoring by persistently acquiring the data of the wearer [9]. Wearable sensors provide reliable and accurate information about human gestures and behaviors to ensure a safe and secure living environment [10]. Gesture recognition is required for the development of various operations such as feedback from acquired data, tracking physical fitness, health monitoring, and self-control/management of a wearable device [11].

Our proposed platform can effectively recognize gestures. Gesture recognition is divided into different steps, the first of which is the collection of data by a body-worn sensor. A tri-axial accelerometer and gyroscope are used with a sampling frequency of 50 Hz. Then, for the removal of noise and unreliable data, pre-processing is performed. The data are then used to extract various time- and frequency-domain features such as entropy, standard deviation, mean, and root mean square values. The features data with labels are then used for classification.

The following were the main objectives in this study:

- Constructing a wearable-sensors-based platform to acquire and recognize ASD children’s gestures.
- Extracting various features from the gestures data and comparing performance to select features for efficient recognition.
- Comparing performance using various machine learning algorithms to increase recognition accuracy.

In the literature related to ASD gesture recognition, the Flex sensor and switch sensors have mostly been used. The limitation of these sensors is that they only have on and off switch status. So, the limited nature of gestures that do not involve much variation in movement is not discussed in the literature. In order to cope with these challenges, the novel contributions in this paper are as follows:

- Since ASD is a special body condition, both medically and physically, we did not use the data of normal people to train the supervised machine learning algorithm for the gestures recognition of ASD. Instead, we collected a novel dataset of 24 physical activities from 10 children who had mild and moderate levels of ASD.
- We performed features extraction on the acquired data using various statistical measures from both the time and frequency domains.
- For gestures recognition on the novel dataset, we evaluated several classifiers and selected the one that produced the most accurate cumulative result.
- We conducted Raspberry-PI-based real-time gesture monitoring of ASD to facilitate the communication between ASD and normal people.

The rest of the paper is organized as follows: Section 2 briefly introduces the related literature work. The methodology is explained in Section 3, and the results and discussion in Section 4. Finally, we conclude the paper in Section 5.

## 2. Background and Related Work

In this section, we briefly focus on the background and the related work conducted regarding the platform used for autism activity and gesture recognition, placement of sensors on the body, extracting features from the acquired sensors data, and the performance comparison analysis of classifiers.

In the literature, some studies focused on the ASD subject, their types of gesture used, and how they behave while communicating with others. Autistic children face difficulties in conveying their thoughts to others. They use fewer communicative gestures compared with typically developing (TD) children [7]. Over time, their parents may grow accustomed to their gestures and understand what their child’s is thinking, but it often remains hard for other people to understand their gestures. Their sign language is normally different from those who are deaf and mute, making it even more difficult to grasp their thoughts. Almost all TD children undergo the same procedure of gestures development [12]. Autistic children may either have an unusual behavioral pattern or have a stereotypic behavioral display [13]. Stereotypic behaviors are abnormal gestures that humans make without having an obvious function or purpose [14]. This kind of behavioral pattern elevates the activation level of children with autism. An autistic person may show some of the classic stereotypic behaviors, e.g., hand flapping, head-banging, body rocking, and top spinning [15].

Human gestures can be detected by ambient-environmental sensors or body-worn sensors. Ambient sensors are installed in offices or homes and are stable in nature, so they cannot perform outside the installed area. These kinds of sensor systems are stationary; hence, they are bound in their specific areas. These systems are not known to have the best efficiency as their observation is strictly bound to a limited area. These types of sensors are highly application-specific. Due to this issue, wearable or mobile monitoring sensors are usually preferred to acquire data continuously and effectively. Mobile monitoring sensors can be used to acquire data remotely and accurately. Despite these many benefits, mobile-sensors-based acquisition has some drawbacks as well [16]. One of them is that most of the time, the smartphone is placed in some pocket position, which reduces the efficiency of recognition of certain activities. Wearable sensors are used to overcome the localization problem. Modern smartphones and smartwatches are equipped with sensors. Gyroscope, accelerometer, magnetometer, temperature, and sound sensors have been used for activity detection [17]. Microphones and web cameras have been used for gestures and expression recognition [18]. A bone conducting speaker, a heads-up display, and wearable glasses were used for recognizing facial gestures [19]. A multisensor accelerometer was used for the detection of stereotypical motor movements (SMMs), which include complex hand movements, body rocking, and mouthing [20]. The Kinect and Flex sensors with a camera have been used to recognize head and hand movements [21,22,23,24,25]. Force sensitive resistor sensors (FSRs) were used to identify multiple gait cycles during walking [26]. Wi-Fi and GPS systems were used for movement recognition [27]. Leap motion device was used to record gesture movements of users for American sign language in virtual reality by generating a 3D hand model [28]. An electromyography sensor (EMG) was used to acquire hand gestures from 15 people. The hand movements included open and closed hand, neutral, victory sign, wrist flexion, tap, and wrist extension. [29]. The Myo Arm band was used to collect gestures data for Hand Cricket between two persons [30].

Sensor placement on human body considers the relative position of the body and the orientation of the sensor. Studies showed that the location of a sensor on a part of the body depends on the activities to be recognized. Accuracy is proportional to the number of sensors used: more sensors lead to higher accuracy and less sensors result in lower accuracy. Researchers have placed sensors on various parts of the body to recognize different activities, such as the wrist, ankle, and chest [31,32,33]. 

For gesture recognition, various features are extracted from data sensed by sensors. Feature extraction is an important and difficult step in activity recognition. The features that have been extracted by different researchers from the acquired sensors data related to different activities are as follows: the duration of activity, mean, variance, standard deviation, median absolute deviation, zero-crossing, cross-correlation, autocorrelation, maximum peaks, total peaks, average of all peaks, area of signal magnitude, energy of signal, frequency component with prime magnitude, mean of the frequency components, power spectral entropy, root mean square, fast Fourier transform, etc. The most commonly adopted classifiers include the k-nearest neighbor (KNN), random forest (RF), multilayer perceptron (MLP), support vector machine (SVM), decision tree, and artificial neural network (ANN) etc. Estrada used KNN and decision trees to recognize static gestures, and the dynamic time warping (DTW) algorithm for the recognition of dynamic gestures [24]. Sombandith et al. [25] used the histogram of oriented gradients and correlation coefficients to recognize the hand gestures of the Lao alphabet sign language. Gonçalves proposed the detection of stereotype movements of autistic people using the dynamic time warping algorithm [22]. Rad used a convolutional neural network for the detection of SMM using a accelerometer sensor [20]. Comprehensive details and a comparison of the proposed technique with techniques reported in the literature are tabulated in Table 1. We investigated gestures or activity recognition based on the type of activities performed, the data set, and the machine learning algorithms applied. 

## 3. Proposed Wearable-Sensors-Based Platform for Gesture Recognition of Autism Spectrum Disorder Children

**Figure 1:**
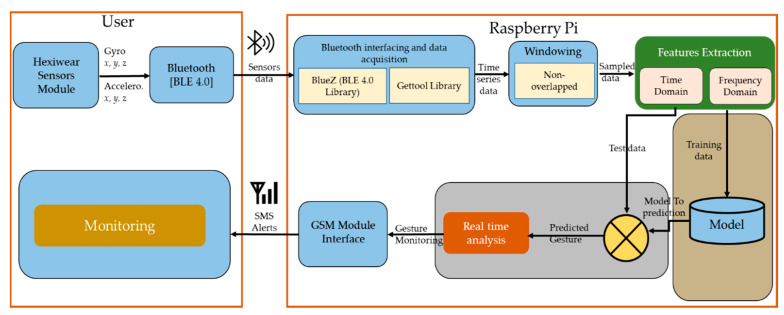
The proposed architecture for wearable-sensors-based platform for the gesture recognition of autism spectrum disorder children using machine learning algorithms.

In this section, we describe the proposed platform, the acquisition of data from sensors, the construction of feature vectors, and the classifiers used for gesture recognition. Figure 1 shows the complete architecture of the proposed framework, which consists of two main parts. 

The first part is based on the acquisitioning of data from sensors and its pre-processing, and the other part consists of the recognition of the processed data using different machine learning algorithms. A single Hexiwear sensor module consisting of both an accelerometer and a gyroscope was installed at the writing position of either the right or left hand. Sensor placement on a child mainly includes the relative position of the activity being performed and the orientation of the sensor. The proposed methodology is explained in the following subsections.

### 3.1. Data Collection

**Figure 2:**
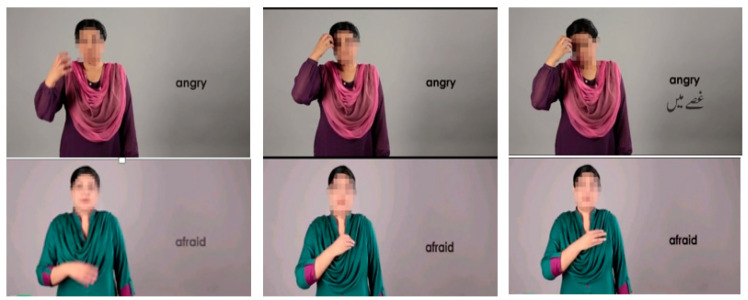
Pictorial overview of the sequence of images for showing the hand movements when performing the gestures.

In the literature related to ASD gesture recognition, the Flex sensor or switch sensors are most often used. These sensors only have on and off switch status. So, few gestures that do not have movement variations are mentioned in the literature. In this study, we collected data from the sensors installed on the wrist of the child. The sensor was worn by the user and data were measured through a specific Bluetooth range of approximately 100 m. The data through sensors were collected at different sampling rates based on sensor type. We configured the sensor by fixing the accelerometer and gyroscope sampling frequency or sampling rate to 50 Hz to precisely capture the hand movement of the gesture. Figure 2 shows a sequence of pictures for two gestures. The gesture signal was digitized and the acquired data points comprise a timestamp and three axes values for the accelerometer and gyroscope. Table 2 shows the sensors configuration that was used for collecting data. The data set comprised different records gathered from the 10 subjects using two sensors. Each subject produced gestures for 24 activities and data were gathered for 3 seconds; this process was repeated 7–12 times. Table 3 shows the complete set of activities for which gestures of ASD children were recorded.

### 3.2. Features Extraction and Selection

**Figure 3:**
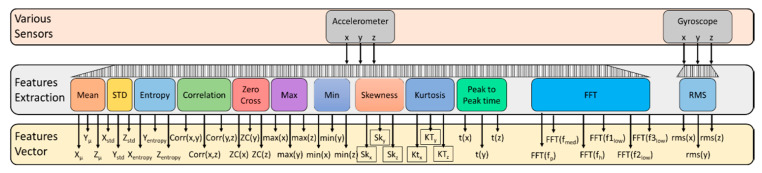
Features vector processing to convert the time-series sensors data into statistical measures in terms of the time- and frequency-domain features.

The collected data were limited to a window size of three seconds, a set of features was extracted from the window, and a specific label was given to this features set, which was then used for learning purposes to construct a trained model. We extracted several features in both the time and frequency domains, inspired by the literature and [48]. The details and formulation of some of the features are given below, and the overall features vector processing is shown in Figure 3.

- **Mean:** We found the mean value for the accelerometer (x,y,z), and magnetometer (x,y,z).

(1) $$\mu =\frac{1}{N}\overset{N-1}{\underset{i=0}{\sum }}x_{i}$$

- **Standard Deviation:** We calculated the spread in the sensors data around the mean as,

(2) $$\sigma =\sqrt{\frac{1}{N}\;}\overset{N-1\;}{\underset{i=0}{\sum }}{\left(x_{i}-\mu \right)}^{2}$$

- **Entropy:** Entropy was used to differentiate between the gestures of a static nature, i.e., low movement, and activities having higher variation.

(3) $$\mathrm{Entropy}=-\frac{1}{N}\;\overset{N-1}{\underset{i=0}{\sum }}p_{i\;}\log p_{i}$$

- **Cross-correlation** was used to help differentiate between activities with variation.
  (4) $$\mathrm{Corr}=\frac{\mathrm{Cov}\;\left(x,y\right)}{\sigma_{x}\sigma_{y}}$$
  where $\mathrm{Cov}\;\left(x,y\right)=\frac{\sum_{i=0}^{N-1}(x_{i}-\mu_{x})\left(y_{i}-\mu_{y}\right)}{N-1}$

- **Zero-crossing (ZC):** Zero-crossing is the number of times the signal crosses zero and its sign changes. We considered ZC for the accelerometer along three axes. Mathematically, it can be written as:

(5) $$\mathrm{ZC}=\mathrm{COUNT}\;(\{(x_{i}>\;0)\;\mathrm{AND}\;(x_{i+1\;}<0)\}\;\mathrm{OR}\;\{(x_{i}<0)\;\mathrm{AND}\;(x_{i+1\;}>0)\}),\;0\;\leq \;i\;\geq \;N\;-1$$

- **Maximum Value:** We calculated the maximum value of the accelerometer (x,y,z).

(6) $${\mathrm{Acc}}_{\max}=\;\max\;(x_{\phi }),\;0\;\leq \;i\;\geq \;N\;-1$$

- **Skewness:** The coefficient of skewness is a measure of the degree of symmetry in the variable distribution. It was calculated for every axis of the accelerometer.

(7) $$\mathrm{Skewness}=\frac{\sum_{i=0}^{N-1}{\left(x_{i}-\mu_{x}\right)}^{3}}{\left(N-1\right)\sigma_{x}^{3}}$$

- **Kurtosis:** The coefficient of kurtosis is a measure of the degree of tail in the variable distribution.

(8) $$\mathrm{Kurtosis}=\frac{\sum_{i=0}^{N-1}{\left(x_{i}-\mu_{x}\right)}^{4}}{\left(N-1\right)\sigma_{x}^{4}}$$

- **Fast Fourier Transform:** Equation (9) was used to find the fast Fourier transform (FFT) of acceleration data. We considered six frequency domain features based on the FFT of the acceleration data. The six features were the FFT magnitude: peak_ f, low_ f 1, low_ f 2, low_ f 3, med_ f, and high_ f.

(9) $$H\left(k\right)=\overset{N-1}{\underset{n=0}{\sum }}x\left(n\right)e^{-j2\pi \left(k\frac{n}{N}\right)}$$

### 3.3. Classification Algorithms for the Proposed Work

The task of this recognition system is the labelling of the recorded gesture from G1 to G24. For this, we used different supervised machine learning algorithms commonly known as classifiers. The process consisted of two parts. In the first phase, the classification algorithm found the relationship between the features and their corresponding labels using the training data to generate a model. Then, in the second part, the model was tested by providing new input features that are unknown to the model and then the model-output labels were compared with the actual labels to determine the classification accuracy of the algorithm. In this study, we used four classifiers, KNN, DT, RF, and the back-propagation model of a neural network. The details of the algorithms are explained in the following subsections.

#### 3.3.1. The K-Nearest Neighbor Algorithm 

The KNN algorithm is known as a lazy method of learning, which means that learning (finding the relationship between input features and their labels) does not start until a testing input is used. The algorithm only finds the k labels from the training data that are similar to the testing input [49]. These k samples and their corresponding labels are then used to predict the label for the new testing input. The closeness was found in terms of Euclidean and Manhattan distances between the new sample and every sample present in the training set in this paper. Equations (10) and (11) were used to find these closeness distances, respectively.

(10) $$D\left(x,y\right)=\sqrt{\overset{n}{\underset{i=0}{\sum }}{\left(x_{i-}y_{i}\right)}^{2}}\;\;\;$$

(11) $$\;\;D\left(x,y\right)=\overset{n}{\underset{i=0}{\sum }}\left|x_{i-}y_{i}\right|$$

#### 3.3.2. The Decision Tree Algorithm 

Decision tree (DT) is a supervised Learning algorithm mostly used to solve classification problems [50]. The main idea is to create a tree for all the data, and process a single outcome at every leaf node or minimize the error at every leaf node. In this structure, internal nodes represent the features of a dataset, branches represent the decision criteria, and leaf nodes represents the outcome. The algorithm uses entropy (E) and the Gini Index (G)-based information gain (I) to select the root node and leaf node. If a number of classes are represented by C, an attribute by A, and  V  represents the possible values in the attribute  A, then the following equations can be used to find the E, G, and I of entropy, respectively.

(12) $$E\left(C\right)=-\overset{C}{\underset{i=1}{\sum }}p_{i}\log_{2}p_{i}\;\;$$

(13) $$G\left(C\right)=1-\overset{C}{\underset{i=1}{\sum }}p_{i}^{2}$$

(14) $$I\;\left(C,\;A\right)\;=E\left(C\right)-\underset{V\;\in \;\;values\;\left(A\right)}{\sum }\frac{\left|C_{v}\right|}{\left|C\right|}\;E\left(A\right)\;$$

**Figure 4:**
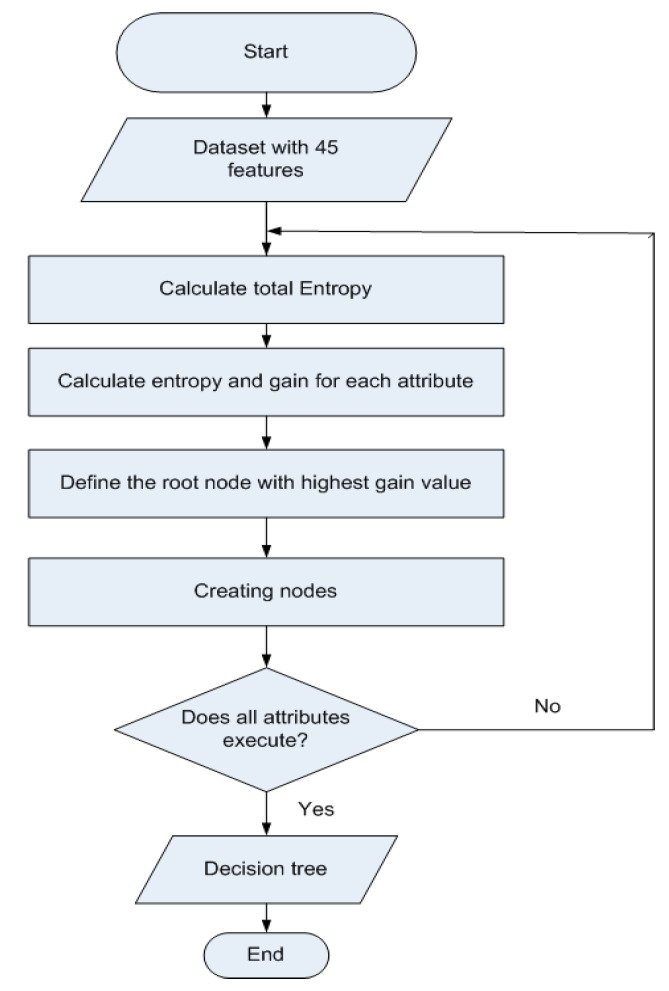
The decision tree algorithm used for the classification of the ASD children’s gestures.

The algorithm maximizes the information gain value, and the node having the highest gain splits first. The algorithm we used is shown in Figure 4.

#### 3.3.3. The Random Forest Algorithm 

Random forest [51] is type of classification that works by building multiple decision trees (weak learners) and finally identifying the decision made by the majority of weak learners. Normally, pruning of the decision trees is used to avoid over-fitting. Pruning is basically a trade-off between accuracy and complexity. No pruning results in high complexity, larger time consumption, and higher resource utilization. Random forest has the same parameters as a decision tree classifier. However, it grows each tree on an independent bootstrap sample from the training data. At each node, a subset of variables is randomly selected from all possible variables (independently for each node) and the best split is found on the selected subset variables. After the forest is formed, the trees are voted or averaged to obtain predictions.

#### 3.3.4. Back-Propagation-Based Neural Networks Algorithm 

The back-propagation model [52] is the core of the neural network training process. It is a method of fine-tuning the weights of a neural net based on the error rate obtained in the previous iteration. Tuning the weights properly ensures the model is reliable by increasing its generalization and reduces the error rates. The feature vector acts as the input to the neural network. Different activation functions are compared to generate the output and then the error is calculated for the back-propagation procedure. The following equations show how the process starts, error is calculated, and the backward propagation with corresponding weight adjustments. Equation (15) calculates the forward value of the input to the output.

(15) $$\;\left[\begin{array}{c} v_{1}\\ v_{2}\\ \vdots \\ v_{n} \end{array}\right]=\left[\begin{array}{cccc} w_{11} & w_{12} & \ldots & w_{1\;45}\\ w_{21} & w_{22} & \ldots & w_{2\;45}\\ \vdots & \vdots & \vdots & \vdots \\ w_{n1} & w_{n2} & \ldots & w_{n\;45} \end{array}\right]\left[\begin{array}{c} x_{1}\\ x_{2}\\ \vdots \\ x_{45} \end{array}\right]+\left[\begin{array}{c} b_{1}\\ b_{2}\\ \vdots \\ b_{n} \end{array}\right]$$

Error calculation:

(16) $$\;\delta =\phi^{\prime }\left(v\right)e\;\;\;\;\;\;\;\;$$

We used the sigmoid function as our activation function, which is given by Equation (17).

(17) $$\;\varphi \left(v\right)=\frac{1}{1+e^{-v}}\;$$

(18) $$\;\varphi^{\prime }\left(v\right)=\varphi \left(v\right)\left(1-\varphi \left(v\right)\right)$$

Error propagation:

(19) $$e^{\left(k\right)}=W^{T}\delta \;$$

(20) $$\delta {\;}^{\left(K\right)}=\phi^{\prime }(v^{\left(k\right)})e^{\left(k\right)}$$

Weight adjustment:

(21) $$\Delta w_{ij}=\alpha \delta {\;}_{i}x_{j}$$

(22) $$w_{ij}{\;}^{new}=w_{ij}^{old}+\Delta w_{ij}$$

where *x* is input, *e* is the error, *v* is the product of weights and corresponding inputs, *W* is the weight matrix, *b* is the bias vector of the node, *ϕ*(*v*) represents the activation function, *ϕ*′(*v*) shows its derivative, α is the learning rate, and i and  j are the output and input node numbers, respectively.

## 4. Simulation Results and Discussion

In this section, we briefly introduce the sensors’ response in time-series, describe the data set, and compare the performance comparison of the machine learning algorithms using cross-validation, i.e., dividing the whole dataset into percentage of training and testing, and leave-one-person-out cross-validation *(LOOCV)* Nine ASD children were used for training and one for testing. 

### 4.1. Sensors Response and Dataset Description

**Figure 5:**
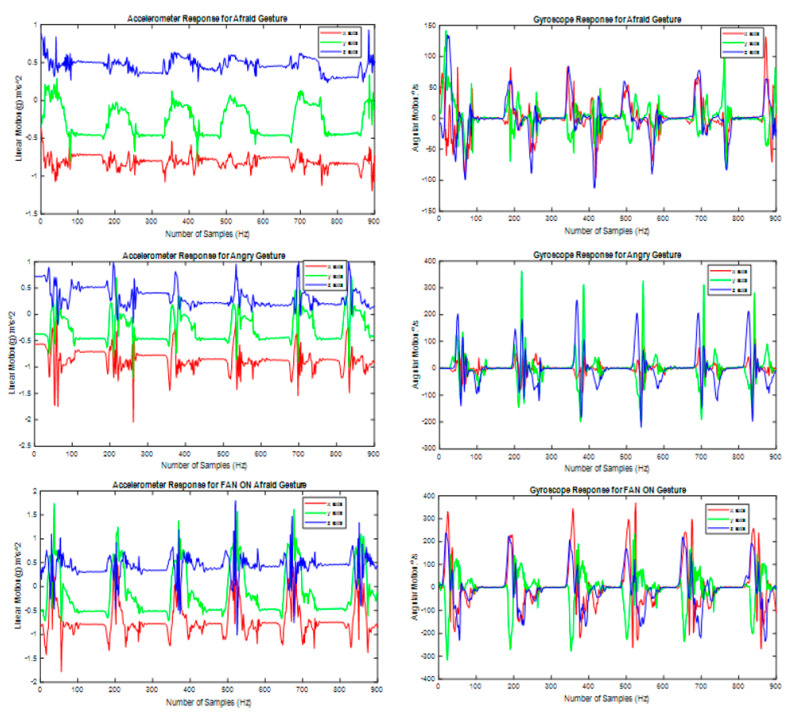
Sensors response for gestures performed by the ASD children (performed six times).

Figure 5 shows the time-series response of the accelerometer and gyroscope for three activities G12 (Afraid), G13 (Angry), and G17 (FAN ON). The sampling period was 50 Hz and the graph shows each gesture was performed six times by the ASD child. Figure 5 depicts that each gesture was performed in 3 s including the variation in hand gesture movement. So, a window of 3 s was used to extract the features for each gesture from sensors data and label it. Table 4 describes the complete data set of the ASD children who performed each gesture 7–12 times so the records for each gesture varied from 83–103 records.

### 4.2. Individual Classifier Performance Comparison Using Data Cross-Validation 

For different classifiers, we used different evaluating functions. For KNN, we used 10 folds for validation and comparison was performed on the basis of distances, i.e., Euclidean and Manhattan. For DT and RF, we calculated both the information gain and Gini index for the different number of trees and variable depths of trees. For the neural network, we compared the results on both the single layer and double layers with a variable number of neurons and learning rates.

**Figure 6:**
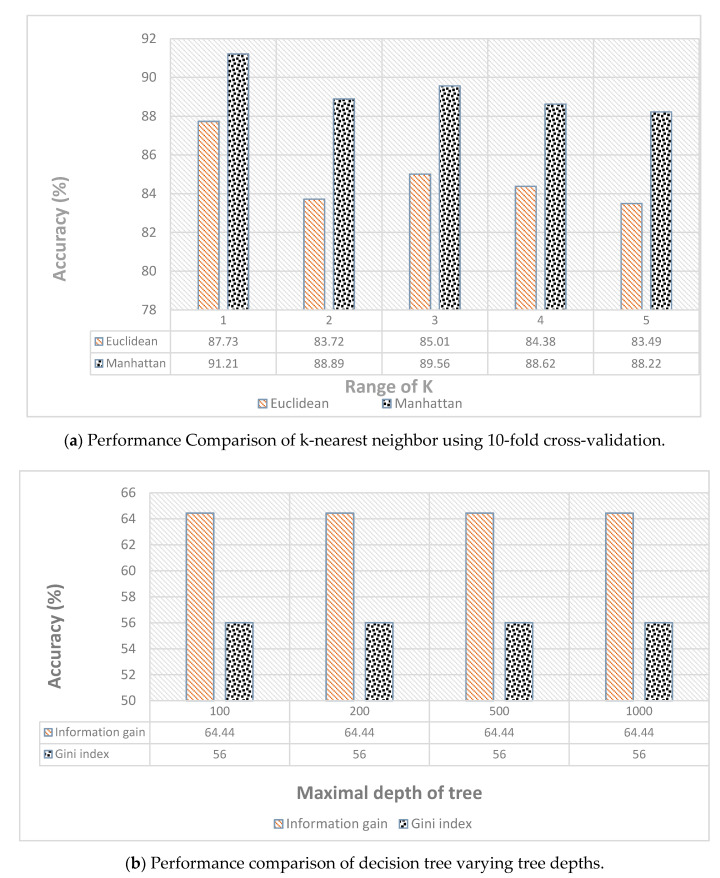

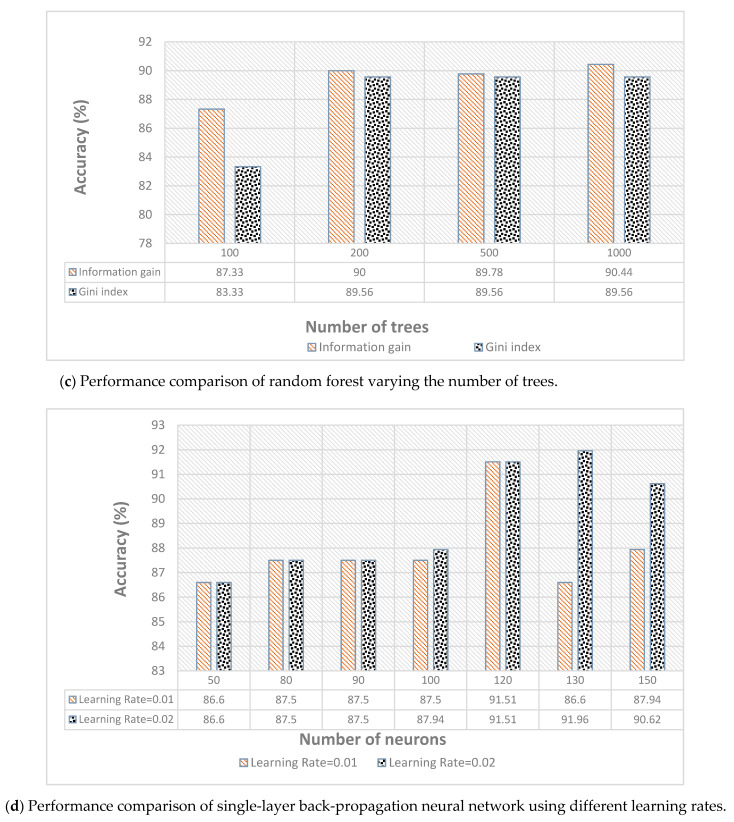
Individual performance comparison of all the classifiers: (**a**) KNN with different distances applied, (**b**) DT (**c**) RF, and (**d**) single-layer NN.

Figure 6 shows the complete individual comparison of all the classifiers using different parameters. Figure 6a compares the performance of the KNN classifier, which clearly shows that the Manhattan distance performed better compared with the Euclidean distance. We used 10-fold cross-validation, i.e., 90% of the data set was used for training and 10% for testing. The accuracy indicates the average of the 10-fold cross-validation. Figure 6 compares the performance of decision tree with varying depths of the decision tree; the data were split into 90% training and 10% testing. The accuracies were found by changing number of trees and the tree depth to evaluate the accuracy. The results are summarized in Figure 6c for random forest. The classifiers performed with accuracy of about 91%.

The results for the single-layer network are summarized in Figure 6d with varying numbers of neurons and using the sigmoid activation function. It achieved the highest accuracy of 91.96% with 130 neurons. At each iteration, the learning rate helps to find the step size needed to minimize the loss function in order to obtain the best parameter that produces the highest accuracy. In the Figure 6d, the learning rate of 0.02 achieved the highest accuracy.

**Figure 7:**
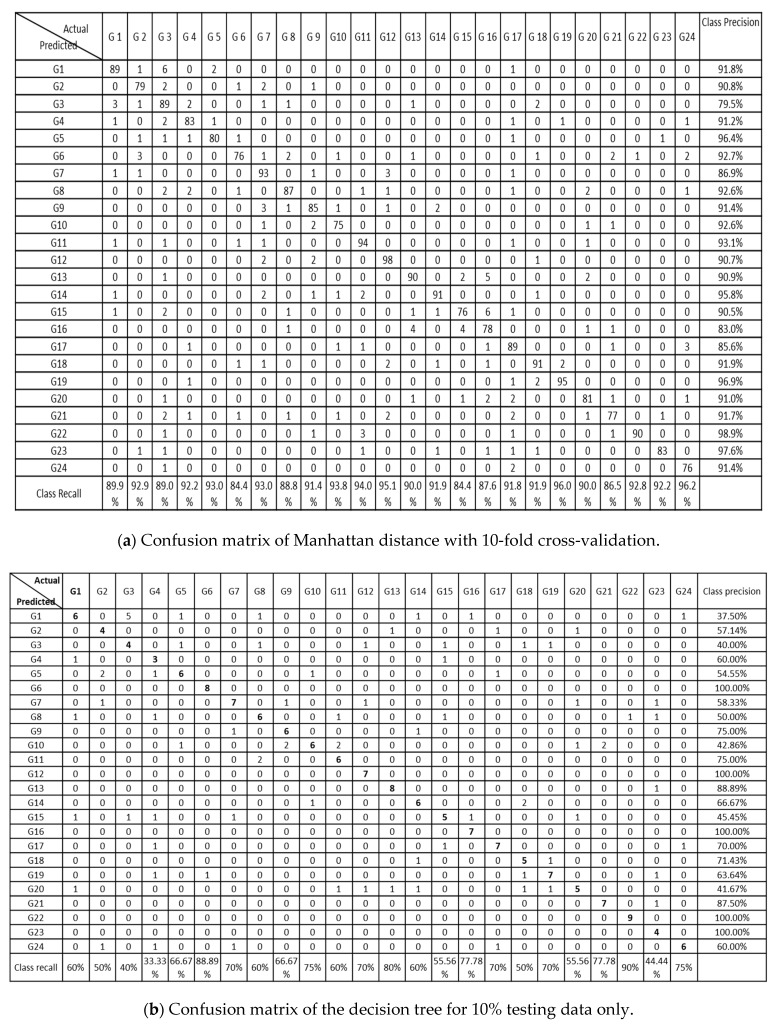

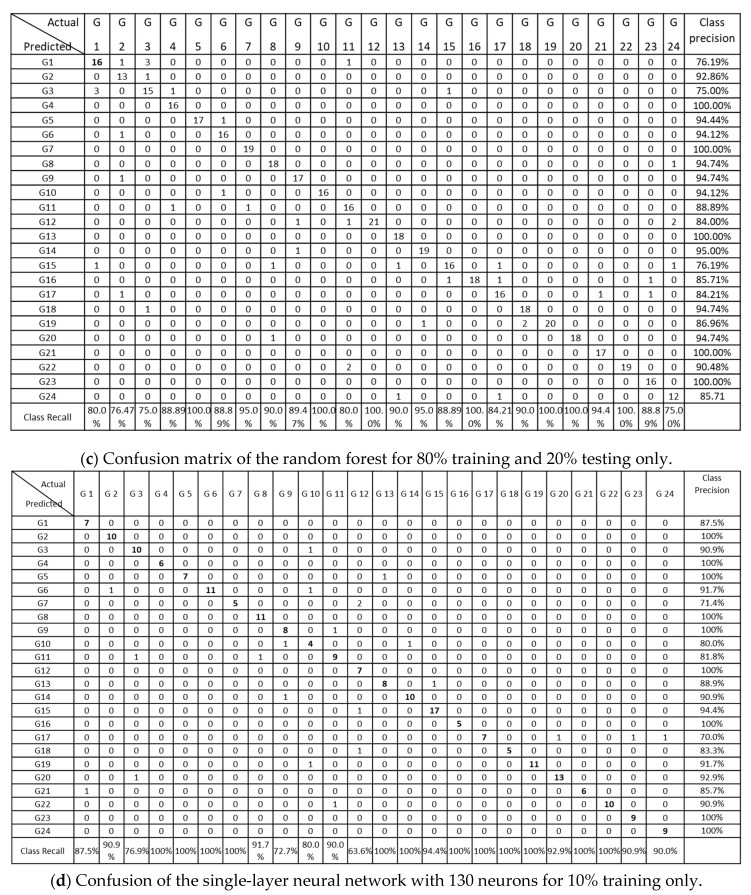
Confusion matrices of all the classifiers: (**a**) KNN, (**b**) DT (**c**) RF, and (**d**) single-layer NN.

Figure 7 shows the confusion matrices for the different algorithms. In most of the algorithms, each individual gesture was recognized with accuracy more than 85%. Due to the similar nature of some gestures, some showed high correlation to each other such G1 and G3, as shown in Figure 7a,c. G3 showed low precision and recall values and had high correlation with G1 due to the similar variation in hand gesture movement. G22, G23, and G24 received the highest accuracy for almost every classification algorithm. These gestures involve angular hand movements that are mostly different from the other gestures. 

### 4.3. Performance Comparison of the Classifiers

**Figure 8:**
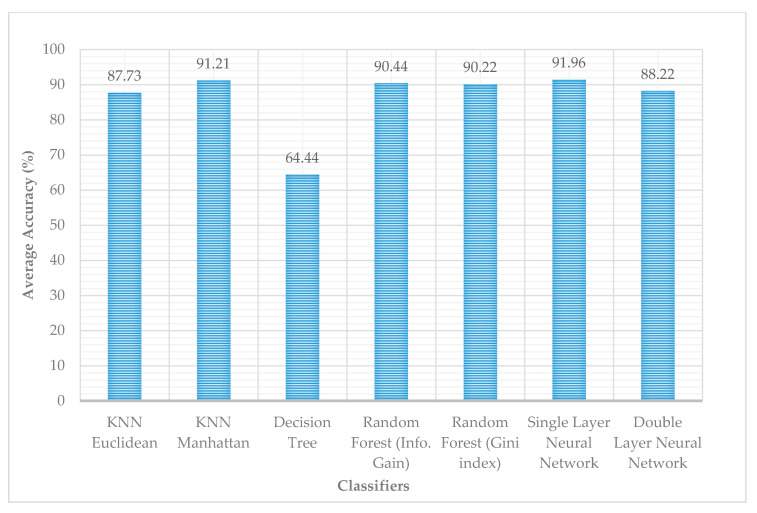
Performance comparison of different classifiers in terms of the accuracy of ASD gestures recognition.

**Figure 9:**
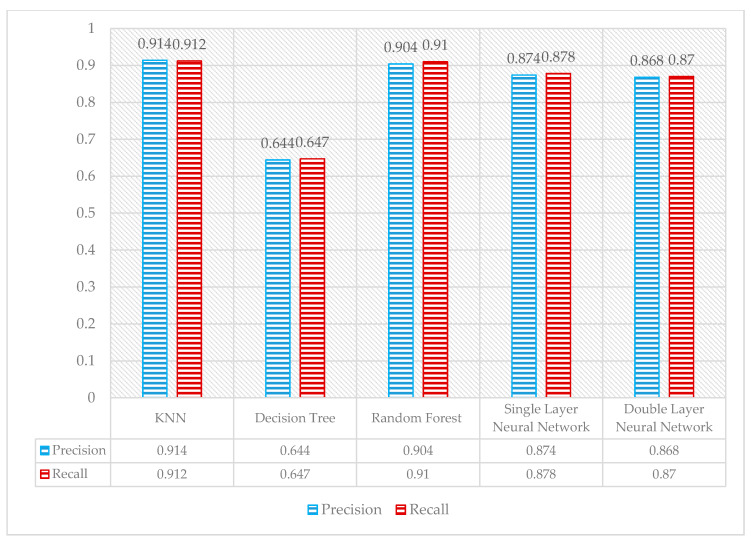
Average comparison of precision and recall values of all classifiers.

The overall comparison showed that the single-layer neural network produced the most accurate results. However, KNN algorithm with the Manhattan distance along with random forest also produced similar results for the data set, as shown in Figure 8. Figure 9 shows the precision and recall of the classifiers. Overall, the three classifiers, KNN using Manhattan distance, RF, and single-layer NN, had an accuracy of about 91%. 

### 4.4. Performance Comparison of the Classifiers Using Leave-One-Person-out Cross-Validation 

**Figure 10:**
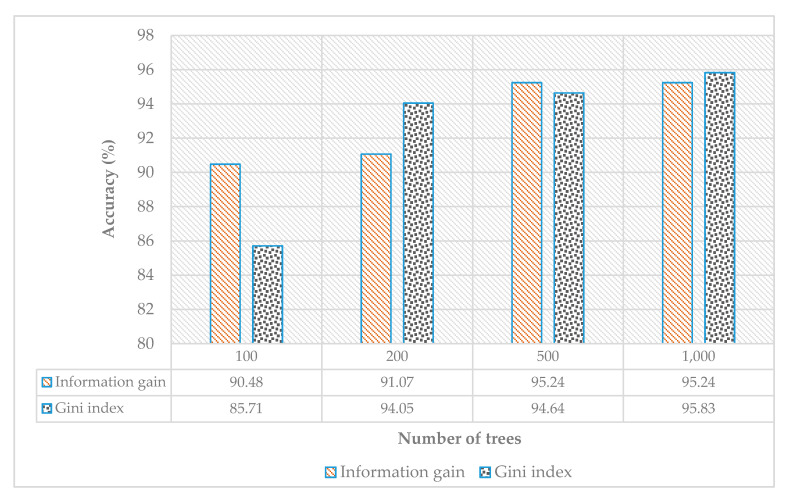
Performance of gestures recognition accuracy of random forest using LOOCV.

In the leave-one-person-out cross-validation (LOOCV), we used the nine subjects for training and one subject for testing. The subject used for testing performed each gesture seven times. We present the results of RF and NN using backpropagation. Figure 10 shows the performance comparison of the LOOCV for RF, which shows that RF using information gain had a recognition accuracy greater than 91%, which was achieved by the 10-fold cross-validation. 

**Figure 11:**
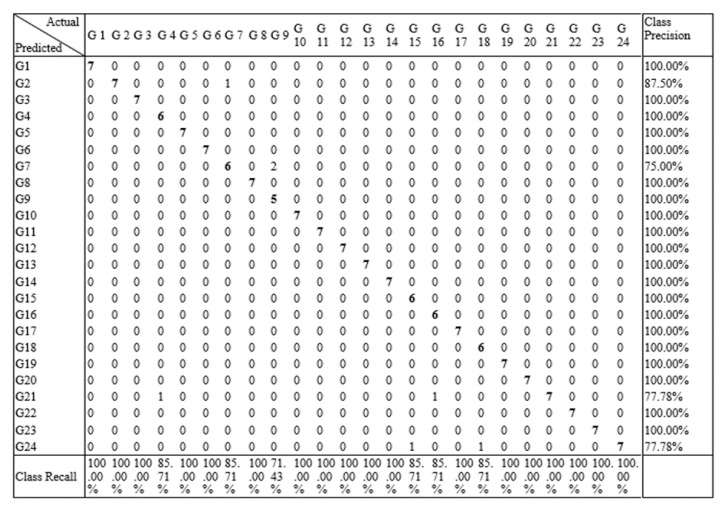
Confusion matrix of random forest using LOOCV.

Figure 11 shows the confusion matrix of RF using LOOCV, which shows that each gesture was recognized with higher accuracy. 

**Figure 12:**
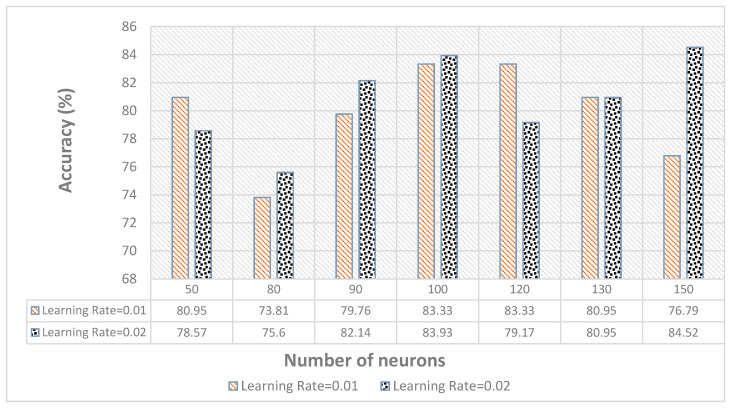
Performance of gestures recognition accuracy of the neural network using LOOCV using different learning rates.

**Figure 13:**
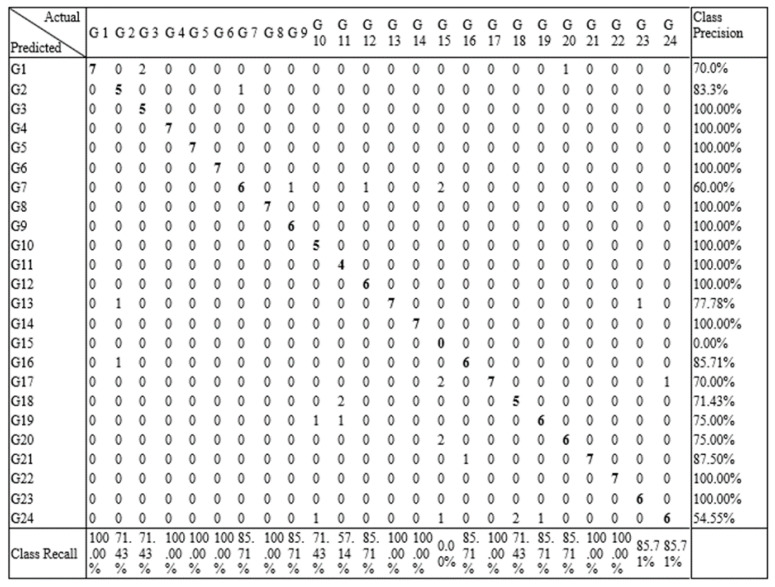
Confusion matrix of the neural network using LOOCV.

Figure 12 shows the accuracy of ASD children gestures recognition of NN back-propagation using LOOCV. The NN also performed better and each gesture was recognized with higher accuracy, as shown in Figure 13 for  a learning rate of 0.02.

## 5. Conclusions, Limitations, and Future Work

In this paper, we proposed a wearable-sensors-based platform for recognizing the gesture movements of children with autism spectrum disorder (ASD) using machine learning algorithms. This work focused on recognizing the daily gestures of ASD children to enable them to communicate with normal people without any hesitation. Modern Androids and smart watches are equipped with sensors such as gyroscopes, accelerometers, and GPS. Smartphones are the most widespread platform used for the recognition of human physical activities. However, their placement on the body creates some limitations, whereas wearable sensors can be placed easily on the human body. We used a wrist-worn sensors module consisting of an accelerometer and gyroscope for the x, y, and z axes and acquired the data at 50 Hz to obtain both the linear and angular motion for increased recognition accuracy of complex gestures. Twenty-four gestures were performed by ten subjects, and each gesture was repeated 7–12 times. A window of 3 s was used to extract various statistical measures (45 features) from the sensors data. The gesture was completed in about 3 s, so we considered a window of 3 s. We compared four classifiers: K-nearest neighbor (KNN), decision tree (DT), random forest (RF), and neural network (NN) using back-propagation using data-based cross-validation and leave-one-person-out cross-validation (LOOCV). Both the data-based 10-fold cross-validation and LOOCV produced accuracies greater than 91%. The RF, NN, and KNN showed about similar accuracy. Most of the individual gestures were recognized with accuracy greater than 90% by both data-partitioning-based cross-validation and LOOCV. This paper focused only the recognition of gestures of ASD children, but the proposed architecture can be utilized for remote health monitoring of ASD children. 

The data were collected in a constrained environment where the ASD children performed the gestures in standing positions. The system and data set have not been validated on sensors data from the ASD children in the sitting or any other body position. The Hexiwear sensor has also limited processing and battery power. Its battery needs recharging after a certain time. The data were collected using a single sensor worn at the wrist position, so may not be capable of recognizing complex gestures. 

In future work, we will work on a multiple- and heterogeneous-sensors-based platform for the gesture recognition of ASD children. We are also working with LSTM-based ML algorithms for validation and performance comparison with RF and NN back-propagation. 

## Figures and Tables

**Table 1:** A summary of the literature and related works about gestures and activities recognition of normal and autistic people.

Ref. No	Sensors	Activities	Features	Algorithms and Accuracy
[2]	Moto 360 smartwatch	Flapping, painting, and sibbing	Discrete cosine transform, FFT, variance, bi-spectrum, z transform, entropy	Simple tree, complex tree, linear and gaussian SVM, boosted and bagged ensemble trees Accuracy: 96.7%
[34]	ECG, accelerometer, gyroscope, magnetometer	Walking, climbing stairs, frontal elevation of arms, knees bending, cycling, jogging, running, jump front and back, sitting, relaxing	Mean, standard deviation, and correlation	Mean prediction rate 99.69%, HMM 89.98%, DBN 92.01%, RNN 99.69%
[35]	Not mentioned	9 uniform hand gestures	Not mentioned, total 576 features extracted	SVM 98.72%
[36]	Gyroscope, accelerometer	Hand movements, body movements	Publicly available dataset features	Convolutional neural network 87.1%, KNN 66.1%, SVM 77.1%, fully CN 88%
[37]	Not mentioned	Static and dynamic unistroke hand gestures	Not mentioned	SVM 97.95%
[38]	Accelerometer, magnetometer, gyroscope	Jogging, walking, cycling jumping, running, jump-rope	Mean, standard dev, kurtosis, skewness, range, correlation, spectral energy, spectral entropy, peak frequencies, and cross-spectral densities	SVM 26%, DT 93.24%, KNN 96.07%, RF 97.12%, Naïve Bayes 76.47%
[39]	Accelerometer, strain sensor	Walking, eating	Mean value, standard dev, percentiles, and correlation frequency domain (energy, entropy)	DT 93.15%
[40]	Camera	Gestures of alphabets	Not mentioned	KNN 94.49%
[41]	Flex sensor, accelerometer, camera,	Malaysian sign language gestures	Not mentioned	General algorithm for the data-glove detection system 78.33–5%
[42]	Camera	24 Fingerspelling static gestures	Not mentioned	KNN classifier 87.38%, Logistic regression 84.32%, naïve Bayes classifier 84.62%, support vector machine (SVM) 91.35%
[43]	Leap Motion Sensor	Gestures for greetings, possessive adjectives, colors, numbers, names, etc.	Not mentioned	Hidden Markov models (HMM) 87.4%, KNN+DTW 88.4%
[44]	Accelerometer	Cycling, sedentary, ambulation	Mean, standard deviation, acceleration range	SVM from 88.5% to 91.6%
[45]	Not mentioned	ASL alphabets and basic hand shapes	The number of fingers, the width and height of the gesture, the distance between the hand fingers, etc.	Type-2 Fuzzy HMM (T2FHMM) 100% accuracy for uniform hand images and 95.5% for cluttered hand images
[24]	Flex sensor	Patterns representing: Letters/Words Numbers	Not mentioned	K-nearest neighbor decision tree dynamic time warpinga verage accuracy = 90%
[46]	QA screening method using mobile app	Not mentioned	Age, sex, ethnicity, country of residence, etc.	RIPPER 80.95%, C4.5 82.54%
[47]	Not mentioned Dataset taken from UCL Machine Learning repository	Common attributes like age, nationality, sex, etc.	Not mentioned	SVM 98.30%, KNN 88.13%, CNN 98.30% ANN 98.30%, naïve Bayes 94.91%, LR 98.30%

**Table 2:** Sensors configuration for the collection of data from autistic children.

Sensors	Sampling Frequency (Hz)	Quantization Levels (Bits)	Range
Accelerometer	50	16	±16 gs
Gyroscope	50	16	±2000°/s

**Table 3:** Information about the gestures which are recorded for data collection.

Gesture	Label	Gesture	Labels
Good Morning	G1	Angry	G13
Good Afternoon	G2	Bulb	G14
Good Night	G3	Cricket	G15
Good Bye	G4	Fan off	G16
Thank you	G5	Fan on	G17
Please	G6	Switch	G18
Yes	G7	Milk	G19
No	G8	Need eraser	G20
Wow	G9	Need pencil	G21
Hello	G10	Need toilet	G22
Sleep	G11	Need water	G23
Afraid	G12	School book	G24

**Table 4:** ASD children data set description.

Gestures Label	No. Records	Gestures Label	No. Records
G1	99	G13	100
G2	85	G14	99
G3	100	G15	90
G4	90	G16	89
G5	86	G17	97
G6	90	G18	99
G7	100	G19	99
G8	98	G20	90
G9	93	G21	89
G10	80	G22	97
G11	100	G23	90
G12	103	G24	78

## Data Availability

The data presented in this study are available on request from the corresponding author. The data are not publicly available now and will be uploaded later to the public repository.
